# Topographic Asymmetry Indices: Correlation between Inferior Minus Superior Value and Index of Height Decentration

**DOI:** 10.1155/2018/7875148

**Published:** 2018-10-02

**Authors:** Sherine S. Wahba, Maged M. Roshdy, Ramy R. Fikry, Mona K. Abdellatif, Abdulrahman M. Abodarahim

**Affiliations:** ^1^Faculty of Medicine, Ain Shams University, Cairo, Egypt; ^2^Consultant of Ophthalmology, Al Watany Eye Hospital, Cairo, Egypt; ^3^Faculty of Medicine, Cairo University, Cairo, Egypt; ^4^Staff Physician Paediatric Ophthalmology, King Abdullah Specialised Children's Hospital, Riyadh, Saudi Arabia

## Abstract

**Purpose:**

To investigate the correlation between decentration index (index of height decentration, IHD) automatically calculated by the Pentacam HR software and the manually calculated inferior minus superior (I‐S) value.

**Setting:**

Al Watany Eye Hospital, Cairo, Egypt.

**Methods:**

In a retrospective study, history taking, clinical examination, and rotating Scheimpflug camera scanning (by Oculyzer II equivalent to Pentacam HR) were done to 128 eyes: 82 normal, 24 forme fruste keratoconus FFKC (apparently normal cornea with evident keratoconus in the fellow eye), and 22 keratoconus (KC). All cases of corneal scars or previous corneal surgeries were excluded. The (I‐S) value was calculated manually from 10 points astride the horizontal meridian. The IHD is calculated automatically by the device software 1.17r119.

**Results:**

The mean (±SD) of (I‐S) value in normal, FFKC, and KC eyes were 0.30 ± 0.93, 0.11 ± 2.03, and 4.62 ± 3.89, respectively, and those of IHD were 0.008 ± 0.004, 0.011 ± 0.005, 0.066 ± 0.067, respectively. The two indices were highly correlated (*p* < 0.0001) with a correlation coefficient (*r*^2^ = 0.874). Deduced regression formulae linking the two indices were calculated.

**Conclusions:**

The two topographic decentration indices are highly correlated. Deduced formulae were proposed linking them.

## 1. Introduction

Although clinical diagnosis of advanced keratoconus (KC) is relatively easy with biomicroscopic and keratometric data, it is rather complicated to rule out subclinical KC before refractive surgery. Corneal topography provided a better means of evaluating the morphologic change in patients with early KC [1].

With the introduction of rotating Scheimpflug devices for corneal tomography, many indices have been proposed for discriminating corneas with subclinical KC from normal corneas including curvature, pachymetric, elevation, and, most recently, biomechanical indices [2]. The most reputable ectasia risk score system [3] uses the topographic asymmetry decentration index, inferior minus superior (I‐S) value, which is not calculated automatically by the Pentacam HR software. Therefore, the topographic asymmetry decentration indices have a special interest.

The aim of our study was to investigate the correlation between the manually calculated (I‐S) value and the automatically calculated index of height decentration (IHD) by Pentacam software and proposed deduced regression formulae linking the two indices.

## 2. Patients and Methods

This is a retrospective study that included 128 eyes; 82 of these eyes had normal corneas, 24 of them were diagnosed as forme fruste KC (FFKC) (a cornea that has no abnormal finding by clinical examination, curvature, elevation, and pachymetry parameters with evident KC in the fellow eye) [4], and 22 as KC. The approach taken was through revising the charts looking for recorded history taking and clinical examination notes of candidates for refractive surgeries in our hospital. All cases of corneal scars or previous corneal surgeries were excluded.

Every eye was scanned at least thrice by a rotating Scheimpflug camera (Allegro Oculyzer II equivalent to Pentacam HR, WaveLight, Erlangen, Germany) software version 1.17r119. Each scan included 25 Scheimpflug images. Data were collected from only one scan which had the largest analyzed area, the highest percent of valid data, and good alignment.

**Figure 1:**
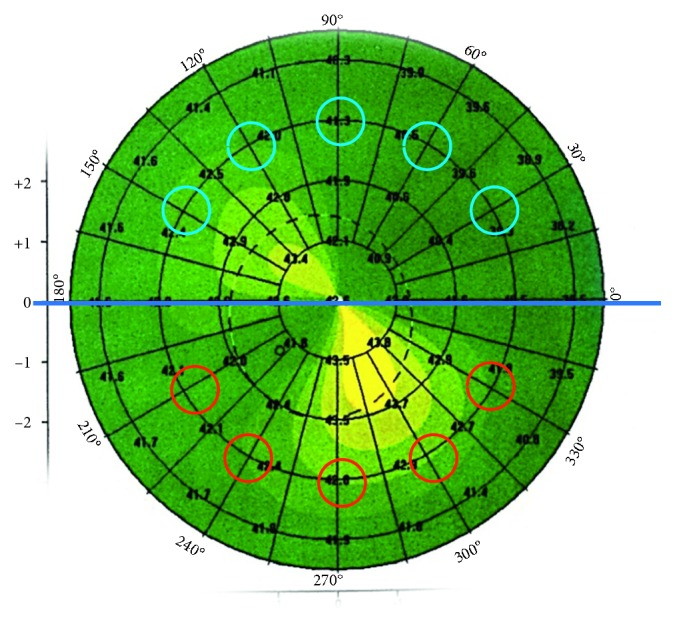
(I‐S) value calculated astride the horizontal meridian.

The investigated indices included the following:

1. Inferior minus superior value (I‐S value) [5]: it is the keratometric dioptric power difference between the average of five points of the inferior hemisphere and the average of five points of the superior hemisphere. It was calculated manually astride the horizontal meridian. The points lie on the 3 mm radius circle at angular intervals of 30 degrees in the axial (sagittal) curvature display as described in Figure 1.
2. Index of height decentration (IHD) [6]: it is the absolute value of decentration of elevation data in the vertical direction (expressed in micrometres). It is calculated automatically by the device.

A validation sample of 78 eyes (52 normal, 10 FFKC, and 16 KC) was then collected prospectively from patients coming for screening or follow up. The right eyes only were chosen except in FFKC due to the paucity of these cases.

## 3. Statistical Analysis

Data were collected, and statistical analysis was performed using MedCalc Statistics 12.5.0.0 (MedCalc Software, Ostend, Belgium). Mean, standard deviation (SD), Mann–Whitney test, area under the receiver operating characteristic (AUROC) calculation and comparison by DeLong et al. method, and Pearson correlation coefficient were calculated. Logistic regression using the enter technique was used to deduce formulae linking the two most correlated indices.

Regarding the validation sample, the AUROC, median, and the 2.5 to 97.5% confidence interval of the absolute difference between the actual and the deduced IHD were calculated.

The study adhered to the Tenets of the Declaration of Helsinki and to the local ethics committee (Watany Research Development Center (WRDC)) standards.

## 4. Results

The mean (±SD) age of the patients was 28.5 ± 7.5 years; 41 eyes of males and 87 of females; 65 were right eyes and 63 left eyes.

The mean (I‐S) value in normal, FFKC, and KC eyes were 0.30 ± 0.93, 0.11 ± 2.03, and 4.62 ± 3.89, respectively, and those of IHD were 0.008 ± 0.004, 0.011 ± 0.005, and 0.066 ± 0.067, respectively.

When differentiating normal from abnormal corneas (KC and FFKC collectively), the two indices gave accurate results (Mann–Whitney, *p* < 0.0001). IHD had the highest AUROC, although no statistically significant superiority of one index over the other was confirmed. The same was found on differentiating normal from FFKC (Table 1).

The two indices were highly correlated (*p* < 0.0001), with correlation coefficient *r*^2^ = 0.874.

**Figure 2:**
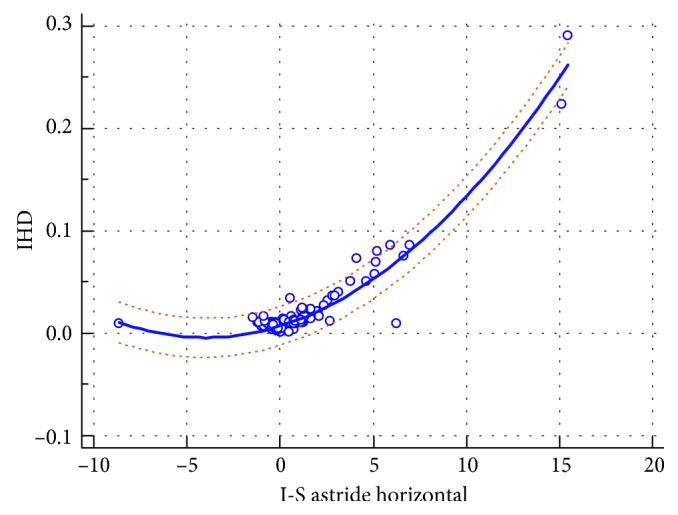
The regression formula deducing the IHD from the (I‐S) value with the 95% prediction lines.

The deduced regression formula linking the two indices (Figure 2) is as follows:

(1) $$\begin{array}{c} \text{IHD}=\text{0}.007334+\text{0}.005676\left(\text{I}‐\text{S value}\right)+\text{0}{.0007021\left(\text{I}‐\text{S value}\right)}^{2}\;\left(r^{2}=\text{0}.9282,\text{residual SD}=\text{0}.009472\right). \end{array}$$

**Figure 3:**
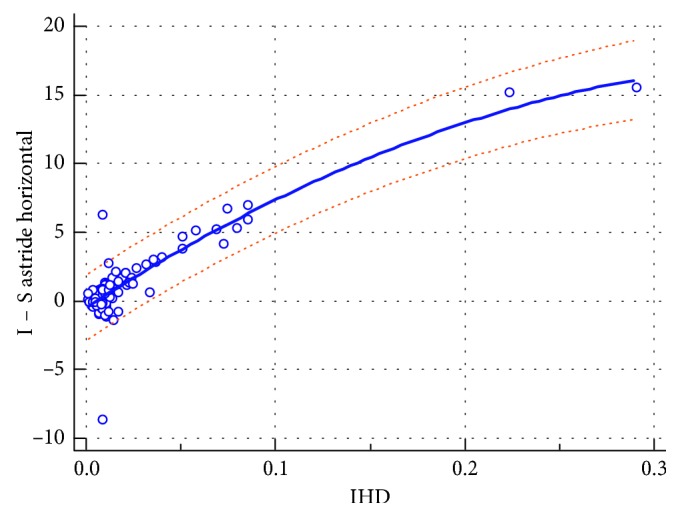
The regression formula deducing the (I‐S) value from the IHD with the 95% prediction lines.

The reverse formula (Figure 3) is as follows:

(2) $$\begin{array}{c} \text{I-S value}=-0.4575+89.5389\left(\text{IHD}\right)-111.8720{\left(\text{IHD}\right)}^{2}\;\left(r^{2}=0.7857,\text{residual SD}=1.1925\right). \end{array}$$

By applying the formula with the inferior half steeper than superior, (I‐S) value of 0.5, 1, and 1.4 correspond to IHD of 0.010, 0.014, and 0.017, taking into consideration that IHD values >0.014 are considered abnormal and IHD values >0.016 are considered pathological [6] (Table 2).

Regarding the validation sample, the IHD, (I‐S) value, and the deduced IHD from the abovementioned formula are represented in Table 3.

The AUROC of IHD and (I‐S) value were not statistically significantly different from each other (Table 4).

The absolute difference between the actual and the deduced IHD is presented in Table 5.

## 5. Discussion

Corneal topography has been found to be sensitive for detecting subtle changes on the anterior corneal surface due to corneal ectatic disorders before the appearance of clinical signs [2]. Rabinowitz [7] had set useful topographic criteria to differentiate between normal and KC suspect eyes. One of these criteria is the curvature asymmetric decentration (I‐S) value with a positive value indicating steeper curvature of the inferior half of the cornea. Value of 1.4 is defined as a cutoff point for suspected KC [8].

Although elevation-based tomography is adding knowledge about the elevation and pachymetry indices of the cornea, still the curvature analysis is very important as the topographic abnormalities are very important evaluated criteria in post-LASIK ectasia and are involved in the ectasia risk score system [3]. The members of the American Academy of Ophthalmology (AAO)/International Society of Refractive Surgery (ISRS)/American Society of Cataract and Refractive Surgery (ASCRS) joint committee [9] recommended avoiding LASIK in patients with asymmetric inferior corneal steepening or asymmetric bowtie patterns with steep axes skewed above and below the horizontal meridian.

Moreover, it is one of the four components involved in KISA% used in KC identification which demonstrated sensitivity and specificity of 96% and 100%, respectively, in terms of keratoconus diagnosis [10].

One of the currently used elevation-based tomography devices is the Pentacam HR; it provides analysis of topometric indices including the automatically calculated decentration IHD index. IHD is not only an index to detect corneal asymmetry, and consequently important in KC detection, but also it was reported as one of the most sensitive and specific criteria for follow up of KC [6].

Our study revealed a high correlation between the (I‐S) value and the IHD.

As the Pentacam software does not include the (I‐S) value, and there is a high correlation between the (I‐S) value and IHD, we deduced regression formulae between them to be able to calculate one from the other with reasonable accuracy. This could enable calculating the Randleman ectasia risk score system directly from Pentacam display, which is currently not possible. We listed the (I‐S) value in normal and KC suspect with the corresponding IHD values as an example.

This regression formula was validated with a small absolute difference between the actual and the deduced IHD. It was higher in KC eyes, mostly due to relative higher vertical asymmetry in these eyes. Therefore, the difference was unlikely to change the diagnosis.

To our knowledge, the current study is the first study to evaluate the correlation between these asymmetric decentration indices and to deduce the formulae linking them.

## 6. Conclusion

The two decentration asymmetric indices are highly correlated, and they can be deduced from each other.

## Figures and Tables

**Table 1:** The AUROC of decentration indices.

Index	Normal versus KC + FFKC			Normal versus FFKC		
	AUROC		AUROC compared to that of IHD (*p* value)	AUROC		AUROC compared to that of IHD (*p* value)
	Mean	SD		Mean	SD	
(I‐S) value	0.768	0.048	0.092	0.582	0.071	0.135
IHD	0.840	0.039	—	0.700	0.061	—

KC = keratoconus; FFKC = forme fruste keratoconus; AUROC = area under the receiver operating characteristic; SD = standard deviation; (I‐S) value = inferior minus superior value; IHD = index of height decentration.

**Table 2:** The corresponding values of IHD.

(I‐S) value	0.5	1.0	1.4
IHD	0.010	0.014	0.017

(I‐S) value = inferior minus superior value; IHD = index of height decentration.

**Table 3:** Validation sample data.

	Median	2.5–97% confidence interval
IHD	0.017	0.003–0.151
(I‐S) value	1.01	0.061–6.475
Deduced IHD	0.014	0.008–0.074

**Table 4:** Validation sample AUROC.

Index	Normal versus KC + FFKC			Normal versus FFKC		
	AUROC		AUROC compared to that of IHD (*p* value)	AUROC		AUROC compared to that of IHD (*p* value)
	Mean	SD		Mean	SD	
(I‐S) value	0.858	0.050	0.710	0.709	0.104	0.648
IHD	0.868	0.048	—	0.735	0.100	—

**Table 5:** The absolute difference between the actual and the deduced IHD.

	Number of eyes	Median of the actual IHD	Median of the absolute difference between actual and deduced IHD	2.5–97.5% confidence interval of the absolute difference between actual and deduced IHD
All eyes	78	0.017	0.004	0.003–0.007
Normal eyes	52	0.015	0.003	0.002–0.005
FFKC	10	0.024	0.007	0.001–0.015
KC	16	0.074	0.032	0.012–0.063

## Data Availability

All data generated or analyzed during this study are included within the article.
